# Communicating Risk for a Climate-Sensitive Disease: A Case Study of Valley Fever in Central California

**DOI:** 10.3390/ijerph16183254

**Published:** 2019-09-05

**Authors:** Melissa Matlock, Suellen Hopfer, Oladele A. Ogunseitan

**Affiliations:** 1 Department of Population Health and Disease Prevention, Program in Public Health; Irvine, CA 92697, USA (S.H.) (O.A.Q.); 2 School of Social Ecology; University of California, Irvine, CA 92697, USA

**Keywords:** Valley Fever, Coccidioidomycosis, behavior adaptation, qualitative research, risk communication, health communication, vulnerability, population health

## Abstract

Valley Fever, or Coccidioidomycosis, a fungal respiratory disease, is prevalent with increasing incidence in the Southwestern United States, especially in the central region of California. Public health agencies in the region do not have a consistent strategy for communication and health promotion targeting vulnerable communities about this climate-sensitive disease. We used the behavior adaptation communication model to design and conduct semi-structured interviews with representatives of public health agencies in five California counties: Fresno, Kern, Kings, San Luis Obispo, and Tulare County. While none of the agencies currently include climate change information into their Valley Fever risk messaging, the agencies discuss future communication methods similar to other health risk factors such as poor air quality days and influenza virus season. For political reasons, some public health agencies deliberately avoided the use of climate change language in communicating health risk factors to farmers who are particularly vulnerable to soil and dust-borne fungal spores. The effectiveness of health communication activities of the public health agencies has not been measured in reducing the prevalence of Valley Fever in impacted communities. Given the transboundary nature of climate influence on Valley Fever risk, a concerted and consistent health communication strategy is expected to be more effective than current practices.

## 1. Introduction

Weather has been known to affect human health [1]. Weather affects the distribution and risk for infectious diseases such as malaria, Rift Valley Fever, plague, Dengue fever, Hantavirus, Ebola hemorrhagic fever, and West Nile virus [1]. Climate and climate change—where climate change is considered the average of daily weather taken across at least 10 years—requires researchers and health professionals to develop new ways of thinking and communicating with the public regarding the health risks associated with the changing climate [2,3]. Many infectious diseases that can be influenced by the climate are vector transmitted diseases. However, some diseases, such as Coccidioidomycosis (Valley Fever), have a sensitive relationship to climate although they are not associated to vector transmission. To date, climate and risk communication researchers are still working on the challenges related to communicating health risks of climate-sensitive diseases like Valley Fever to diverse communities and decision-makers [1].

Effective health communication should inform the public about the negative health effects and actions taken to reduce a community’s risk [3]. There are four guiding principles for health communication initiatives: (1) identify the targeted individual behavior; (2) develop an effective strategy for exposing people to the message; (3) take a comprehensive all-inclusive approach; and (4) seek routine media exposure [4]. In addition, communication around health risks requires an open dialogue with two-way communication with the communities of interest [5]. Health-related climate risk messaging needs to be framed to effectively reach at-risk audiences who may vary depending on language spoken, educational background, or exposure risk [6]. Effective risk communication should focus on process, presentation, and comparing risks [5]. For the process, it is important for health agencies to know their limits, pretest their message, communicate early and often, and to understand that the disease’s perception is also its reality [5]. A successful communication approach will involve empirical research on how climate and health issues are related, scenario development to understand vulnerabilities, and the development of risk-reducing strategies [3]. Data drives risk decision-making [7]. A majority of experts do not realize how poorly they are communicating, where in some situations, uncertainty can come off as evasive [7]. 

Past climate communication focused on the idea of attitudinal change, on getting people on board with the idea of climate change [8]. However, attitudinal change alone will not work [8]. There are other factors that influence the link between individuals’ attitudes and behavior change, such as social norms, government regulation, and removing impediments [8]. Additional limits to adapting to climate and climate change are also contingent on ethics, knowledge, and attitudes to risk and culture, which are endogenous to society [6,9]. It is also important in risk communication to show your audience how they might benefit from an activity that might affect them [7]. 

Another principle of risk communication is focused around presentation [5]. Health agencies need to know their audience and to communicate in their audience’s native language. It is also important to simplify the language but not the content. Tailored messages to specific risk groups are important, but health agencies need to convey the same information to all audiences [5]. Comparing risks through the use of analogies, ranges, and traits is another component of successful risk communication [5]. In addition, according to the World Health Organization, one of the principles for effective communication is to have actionable items for the community to adopt [10]. Health campaigners often design prevention campaigns with messages focused on motivating individuals to avoid certain behaviors that put their health at risk. However, in certain environments, individuals may not be able to avoid risks entirely, particularly when their occupation or home inherently places them at risk [11]. In such cases, avoidance risk messages will likely be ineffective and become overbearing [12]. Cases such as these can lead to trust issues in risk management [13,14]. Climate communication needs to understand an individual’s relationship with their environment [15]. For example, migrant workers will not be able to avoid pesticide and sun exposure or children living in older, poorly resourced homes may not be able to avoid lead-contaminated environments. Similar sentiments have also been shown in environmental hazard management [12]. In these cases, designing messages that encourage behavior adaptation rather than avoidance to one’s given risk environment may afford a greater likelihood of message acceptance [16]. Prevention messages grounded in behavior adaptation inform message design. For example, advocating for wearing sunscreen and hats with sun protective flaps for the neck for individuals exposed to prolonged sun is a behavior adaptation. The concept of adapting health risk messages for audiences who engage in a behavior that threatens their health, to encourage the adoption of specific prevention or detection methods to reduce that harm is explicitly expressed in the behavior adaption model to guide the design of effective health risk messages [11]. By incorporating the behavior adaptation model with risk management, it is important for health agencies to make their audience their partner and give them a seat at the table where the audience can take a more active and constructive role [7]. 

To effectively prevent climate-sensitive diseases such as Valley Fever in at-risk communities, risk messages encouraging behavior adaptation warrant consideration [17]. For those sensitive to poor air quality because of asthma who cannot avoid going outside, adaptive risk messages may signal risk when a threshold has been reached. An illustration of this would be when air quality is rated as “Red” days, suggestive of passing a threshold that must be avoided. The best prevention is to stay indoors during the time of the alert, but disposable respirators known as N-95 or P-100 respirators can be used for outdoor workers [18]. 

How can public health agencies communicate adaptive behavioral responses for Valley Fever that require unrealistic behavior changes to reduce risk? Theoretical developments in climate change communication focused on the role of language, such as metaphors, strategies, frames, and narratives in conveying climate change issues to stakeholders [6,19]. Historically, adaptive responses in communication by organizations have also been influenced by endogenous factors like attitudes to risk, as well as external factors like budget, economy, and institutional priorities [20]. Behavioral adaptation also needs to focus on characterizing, assessing, and conveying uncertainty [14,21].

What can climate communication look like for a disease whose climate relationship is still being researched to disentangle confounding variables like construction activities? Given that Valley Fever is a climate sensitive disease, is there a way to effectively incorporate climate and climate messaging to make more specific behavioral changes for Valley Fever prevention?

### Climate Change and Epidemiology of Valley Fever

Coccidioidomycosis, known as Valley Fever, is a growing public health concern for the Southwestern United States, primarily Arizona and Central California [22,23]. The causative agents *Coccidioides immitis*, or *Coccidioides posadasii*, and their spores are typically found at around 10 cm below soil [24]. Exposure on endemic land typically occurs when the soil gets disturbed and aerosolized. Once the spores are aerosolized and inhaled by humans and animals, they can cause disease. The fungal spores are endemic to the Southwestern United States, Mexico, and parts of South America. In the United States, Arizona has the highest incidence of cases, with approximately two-thirds of all cases reported nationwide and California is the second highest. Although most of the counties in California diagnose people with Valley Fever, Central California has the largest incidence in the state. The Central Californian counties with the highest incidence are Fresno County, Kern County, Kings County, San Luis Obispo County, and Tulare County [25]. 

Listed on the Centers for Disease Control (CDC), Coccidioidomycosis symptoms are similar to the flu: fatigue, cough, fever, headache, rashes, shortness of breath, muscle aches or join pain, and night sweats [25]. Transmission of the disease is from directly inhaling fungal spores from the environment. Person-to-person transmission does not typically occur. For approximately 60% of diagnosed cases, the disease will remit in a few months without the need for treatment [26,27]. However, those with more severe symptoms will typically be treated by their healthcare provider. 

Although typically treated with various antifungals, such as Amphotericin B deoxycholate (0.5–1.5 mg/kg per day), lipid formulations of Amphotericin B, which can be easier to absorb (2–5 mg/kg daily), Ketoconazole (400 mg daily orally), Fluconazole (400–800 mg/day orally), and Itraconazole (200 mg twice per day or three times orally), there is no cure for Valley Fever [26,27,28]. Patients with active coccidioidomycosis and disseminated disease are typically prescribed antifungals for 3–6 months and hospitalizations are common. It is estimated that 10%–50% of those living in endemic areas have been exposed to some form of the fungal pathogen, *C. immitis*, or *C. posadasii*. Each year, it is estimated that approximately 150,000 new cases occur in the United States [29,30]. A majority of the nationwide cases occur in Arizona and subsequently, Arizona has been at the forefront of understanding Valley Fever and risk communication.

Since the 1950s, climatic factors, particularly precipitation but also wind, were considered to have a “Grow and Blow” effect on the *C. immitis* spores [31,32,33,34,35]. The “Grow and Blow” effect hypothesizes that in order for the fungal spores to germinate, there needs to be an increase in soil moisture. Then, a dry period needs to occur to make the soil loose and easily disturbed by wind in order to disperse the spores for inhalation [31,32,33,34,35]. Temperature is also said to have a role in the exposure of these spores. During dry, hot periods, temperature is said to sterilize the topsoil, reducing the competition against the C. *immitis* spores [33,36]. However, analysis of this relationship did not occur until the 2000s. Several of these studies found the roles of climatic factors on incidence to not be fully understood [37,38,39,40,41,42,43,44,45]. 

The highest annual number of Coccidioidomycosis cases (more than 5000) in California were documented in 2016, surpassing the previous year of 2015 by an estimated 34% [23]. It is unclear why this disease incidence increased [23], but new research is attempting to understand the climate’s influence on the disease [45]. 

The purpose of this study was to (a) describe how Central California’s public health agencies communicate Valley Fever risk currently, (b) identify how they adapt Valley Fever risk messages, and (c) what additional information public health agencies perceive as improving disease prevention efforts for eliminating seasonal Valley Fever prevalence.

## 2. Materials and Methods 

### 2.1. Study Location

**Figure 1:**
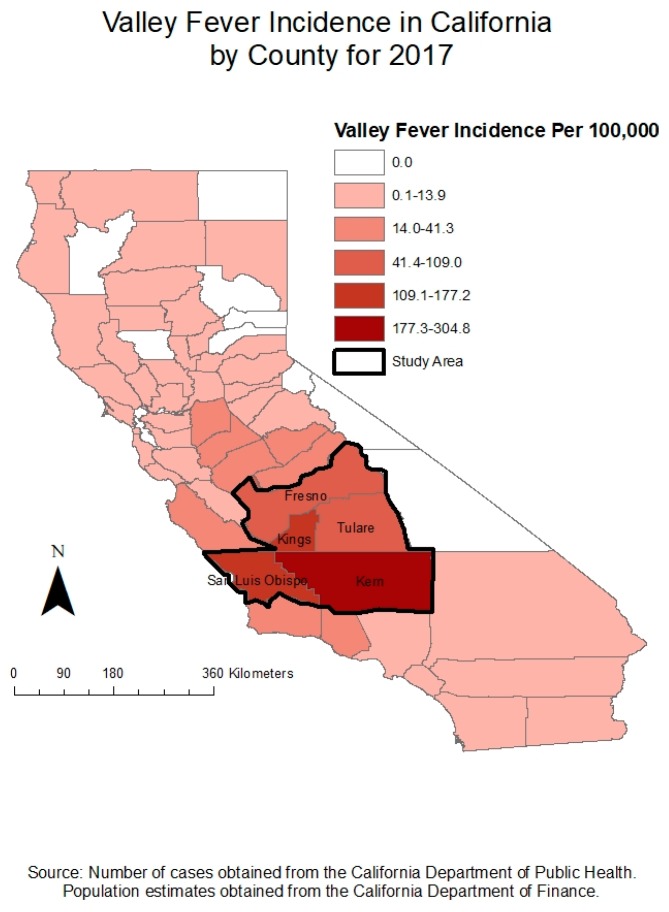
Valley Fever incidence in California by county for 2017.

Five governmental public health agencies from regions in Central California most impacted by Valley Fever cases were recruited for interviews to understand current and desired public health risk communication and prevention messaging employed to address Valley Fever as a public health issue (Figure 1). 

### 2.2. Data Collection

Staff members in a position to be representative of the agency and who have knowledge of the operations, resources, and budget of the agency were purposively recruited for this study. These individuals had titles such as Public Health Director, Assistant Director, Division Manager, Program Manager, and/or Health Officer. At least 1 staff member from each of the 5 Central California public health agencies most impacted by Valley Fever (Fresno, Kern, Kings, San Luis Obispo, and Tulare counties) participated in this study *(n =* 8). Interviews ranged in length between 25 min and 45 min. San Luis Obispo and Tulare counties had more than one person participate in a group interview. No staff members from any of the agencies chose to not participate. The research study protocol was approved by the research university’s Institutional Review Board (IRB). Anonymity was kept by referencing the interviewees as representatives of the county. 

An interview guide was developed to explore the following: (1) How do Central Californian public health agencies currently communicate Valley Fever risk to their communities? (2) How would these agencies like to see climate-related risk factors contributing to Valley Fever risk integrated as part of Valley Fever prevention messages? (3) What challenges do public health agencies perceive with respect to adapting climate factors contributing to Valley Fever risk to their local communities? The interview guide was organized, and questions were grouped to address Valley Fever data, evaluation, and communication. Questions were derived from the Local Public Health System Performance Assessment Instrument. Interviews were semi-structured and conducted over the phone, audio-recorded, and transcribed verbatim. The principle investigator conducted all the interviews. Detailed notes were taken during the interview to verify the transcribed interviews.

### 2.3. Data Analysis

Two coders independently read the transcripts becoming familiar with the data. After data immersion, primary cycle line-by-line coding of the transcripts was first conducted identifying segments of the data as belonging to or representing meaningful phenomena [46]. Data coding was done manually. Primary coding described how public health agencies communicate Valley Fever risk, what kind of information they collect and have, how and when they disseminate risk information, and what their resources are. Second level coding was then conducted to group and organize codes, synthesize and categorize emerging codes into higher order emerging themes, ensure research questions were answered, and analyze data for how Valley Fever risk messages were adapted. Constant comparison was subsequently performed comparing and contrasting data across the five public health agencies [46]. Themes were examined for answering the research questions: How were Valley Fever risk messages communicated? How were messages adapted to existing risk environments? Was climate information integrated? What were continued challenges for communicating Valley Fever risk? Coders examined the data for saturation of codes and discussed emerging themes [47]. Discrepancies were discussed to ensure coding consistency. 

An audit trail of the data includes the transcripts and codebook, which are available upon request [48,49]. Valley Fever is expected to increase over the next coming years and there is little that is known about the disease. Exploring how public health agencies utilize climate data for more accurate risk communication of Valley Fever is a relevant, timely, and worthy topic of research [49]. The original research focus of interviews was to determine how agencies process Valley Fever data, their resources, and how they communicate Valley Fever and climate with the public. In this analysis, the researcher discovered the inclination towards a code related to research and partnerships. With the help of a second coder, the focus of the research changed and allowed for the opening of ideas related to media campaigns and target audiences. 

## 3. Results

### 3.1. Research Question 1: How do Central Californian Public Health Agencies Currently Communicate Valley Fever Prevention to their Communities?

#### 3.1.1. “Get Tested” Valley Fever Prevention Messages

Most public health agencies focused their Valley Fever prevention messages on getting tested if you exhibit symptoms. Tulare County focused on “Just kind of give them education on when the risk is higher. When it’s dusty out. What kind of occupation they have, and such.”

#### 3.1.2. Aligning Dissemination of Valley Fever Risk Campaigns with Peak Seasonal Onset

The timing of “get tested” campaigns for most counties was launched at the end of summer. This coincided with August being Valley Fever Awareness Month. For example, Tulare County launched awareness campaigns during this time “that’s kind of the peak, or the beginning of the peak, of the season with all the dryness and dust.” Fresno County reported two phases of messaging. 

“When we look at our data set, for us, when are we going to actually do TV, do radio, do social media, do all of that, the data drives us to do that and we like doing that around this time of year. We’ll do it right around February, March. We’ll do it around also— I’ve done one in the summertime a little bit but that’s kind of a little bit late and we’ll do it again in I think fall if there’s any funding available.” —Fresno County

#### 3.1.3. Targeted Messaging for At-Risk Groups 

Public health agencies all stated that they target their messaging to at-risk communities. These might include construction workers, farmers, and the prison population. Educational materials were often provided in at least two languages, English and Spanish. However, their approach to messaging differed depending on audience. 

##### Valley Fever Messaging for Construction Workers and Sites 

For construction workers, messaging focused on dust containment and wetting of soil. All counties partnered with their planning departments to hand out Valley Fever awareness documents to construction projects with messaging related to keeping the dust down. Kern County stated, “But we know it’s a known risk and it’s a known hazard in Kern County and that construction and any other of those planning elements needed to take that into account and to take steps for mitigation and for employee education.” 

In San Luis Obispo County, prevention procedures were even integrated as conditional mechanisms of administrative approval on construction projects: “W worked very hard to make sure the conditions of approval for big projects, there is most definitely language inserted into the conditions of approval that talk about high wind days and the need to use water truck to keep the dirt down.”

##### Valley Fever Messaging for Farmers

Prevention messages for farmers included more frequent and routine tilling of soil to prevent excessive fungal spores from growing. Fresno County had a hypothesis that water allocations to the farmers are linked to Valley Fever outbreaks and that prevention for farmers could occur by controlling the moisture in the soils. However, this is an informal hypothesis that has not been researched. 

“Since we have a less stable water allocation, what does that do? Our farmers on our side then aren’t planting crops because their water allocation is unstable. What that means is they’re not tilling the ground, they’re not working the land as much to disturb the potential growth of the spores, so-- of the fungus. So with us, going through years, you’re letting the …ground is not being tilled. And then, we get a wet year. The wet year loves to feed the fungus even more. So that first tilling in your wet year, when everybody’s going back to work. Now, your expectation here is you’re going to give more admission of that fungus out into the air. And so, because of the unstable water allocation, our farmers aren’t tilling the ground as often as they could to actually prevent this.” —Fresno County

In addition to frequent tilling, wearing proper protective equipment during peak risk times was another focus of Valley Fever messaging for farmers. Fresno County, Kings County, and San Luis Obispo County reported these messaging strategies. From the Kings County representative, “We tell them they should be wearing N95 respirators when they’re out there, especially people that are not endemic to the area and come in from other areas.” 

##### Valley Fever Messaging for the Prison Population

Certain living conditions, especially in places of confinement such as prisons, where a special population is concentrated in one location and consequently, has an exacerbated risk for acquiring Valley Fever. Kings County mentioned that they have a high incidence of Valley Fever among correctional facilities. “So a wind comes out of the northwest and goes southeast, right, and that tends to be where our concentration of cases are. And then, of course, and you may have found this out too, but then confinement. Hence prisons. We’ve got three prisons here and then all three it has been an issue.” 

However, the struggle for Kings County is what to do to prevent the high-risk season from being exposed. They are working on it but do not have an answer. Kings County struggled with the messaging to prisons because the prisoners are confined in the endemic zone and avoiding exposure for long-lengths of time is not practical.

#### 3.1.4. Absence of Explicit Discussion about Valley Fever as a Climate-Sensitive Disease

Overall, participants do not currently incorporate climate information into their Valley Fever communication, but they were aware that there is a relationship to climate. To keep messaging simple and avoid information overload, public health agencies had not integrated explicit climate information into Valley Fever messaging. Climate factors such as wind however, were discussed, but were not incorporated into explicit Valley Fever risk messages. 

“But from a climate perspective, we understand that hot, dry weather will promote the spread of cocci when the wind blows. We also are aware of the fact that valley fever does seem to have some linkages to weather change patterns. After a long period of drought, we know that the fungus does not proliferate as well in the soil and so case rate goes down. And then when you get a really wet period like half a couple of years ago, case rate go skyrocketing. That tends to be a definite correlation… We don’t pass along all the research. We really try to keep—we understand that the people we’re trying to reach face an information overload in every aspect of life and so we really try to defer down to simple messages and occasionally a different point of view which is really relevant which shows up sort of as a way to pique interest.” —San Luis Obispo County

#### 3.1.5. Climate Influences on Valley Fever Discussed as Wind Messages

When asked about climate’s relationship to spore growth, all agencies discussed rain. When asked about climate’s relationship to Valley Fever outbreaks, all agencies discussed wind. Public health agencies perceived the scientific evidence correlating fungal growth with Valley Fever cases as too unreliable and not specific enough to use in risk messaging strategies and awareness strategies. 

“We always kind of talk about it. We make the joke after windy days. Like, ‘Well, in a month, we’re going to get a bunch of Valley Fever cases.’ No one’s so far been able to give me a really good sense of predicting Valley Fever and of course, predicting fungal growth doesn’t predict the number of cases you’re going to see. But we like to assume there’s some kind of correlation there.” —Kern County

### 3.2. Research Question 2: How Would these Agencies Like toSsee Climate Communication in Relation to Valley Fever?

Two main themes arose: metaphor communication and risk communication. The biggest challenge the agencies experienced with communicating climate information was how to apply that information into messaging that does not result in message fatigue and target audiences discounting public health Valley Fever risk messages.

#### 3.2.1. Comparisons with Public Health Risk Messages Already Familiar to the Public

Public health agencies discussed communicating Valley Fever risk by drawing analogies to poor air quality days or red flag or “Red” days surpassing thresholds. Target audiences living and working in these counties are already familiar with public health risk messaging about poor air quality and attend to these kinds of messages. One strategy then, is to couple or piggyback Valley Fever messaging jointly with poor air quality risk messaging. This “kills two birds with one stone” so to speak, given the agencies’ minimal resources and the public’s limited attention to health risk messages. Furthermore, Valley Fever risk messaging cannot ask the public to avoid the environment in which they routinely work and live; they can at best, ask people to adapt to their environment to minimize risks to their health. This message concept draws on the behavior adaptation model (BAM) [9]. Kern County suggested “We cannot tell people, ‘Don’t go outside if it’s windy or dusty for the entire Valley Fever season,’ if there is a Valley Fever season. But that’s just really hard for people to maintain.” To address this issue, Kern County made the analogy to poor air quality. 

“What I’d like to find out is what does windy mean? Does that mean winds of 5 mph, winds of 35 mph? So that we could more accurately warm people about their risk of Valley Fever. In my head— I’ve told this to a couple of people I think—the same way we have air quality flags and so this is a red day for air quality. If you’re in a sensitive group, you need to stay indoors. But I’d like to see if we had something like that for Valley Fever. If it was something as simple as correlating it with air quality. That if it’s a poor air quality day, it’s probably a poor day for Valley Fever. That people could use that as a gauge of their risk. Right now, it’s very general if you—if it’s windy or dusty, go inside. But how windy, how dusty?” —Kern County

#### 3.2.2. Analogies with Health Conditions Familiar to the Public

Another form of climate communication of interest to the health agencies employed a message strategy that addresses the uncertainty inherent to Valley Fever and the agencies’ prevention measures. In discussing successful communication strategies for the agencies, all agencies made comparisons between flu and Valley Fever. 

“So, what I would hope for is that we could say, ‘Windy days increase your risk and when the wind is over 50 miles an hour, you’re at increased risk of Valley Fever.’ You should always take precautions but since it’s an increasing windy day, then you might think about it more often. You’re much more likely to do it. Just like when it’s a ‘bad flu season’, people are going to run off to get their flu shot.” —Kern County

Kings County also made a comparison to allergies. “We know this year especially is going to be bad for allergies because of all the rain. We need the rain, but the rains—it’s awesome. But it’s also going to have a collateral effect with the allergies, right? And the same thing with these kinds of a fungus, right? If the temperatures are right, they’re in these spores, well they become active.”

#### 3.2.3. Valley Fever Risk Message Fatigue

When discussing their media campaign, San Luis Obispo County believed their media market is fairly saturated with Valley Fever messaging. For them, “We understand that the people we’re trying to reach face an information overload in every aspect of life and so we really try to defer to simple messages and occasionally a different point of view which is really relevant which shows up sort of as a way to pique interest.” 

For Tulare County, “We certainly don’t do a media release every single month. I think people wouldn’t pay much attention in that case.” Fresno County discussed how messaging needs to be phrased a certain way in order to avoid fatigue in the messaging. 

“’Hey, there’s a windstorm coming,’ that media message will die out so fast. It’s kind of like crying wolf all the time. And so, all it’s going to do is really be— it’d be exciting, and you’ll effect change immediately, but that’s not a sustainable media campaign because we really can’t connect the two, right? And so, is it 20 mile-an-hour winds? Is it 10? Is it 30, or is it 60? Without having not seen the research, that’s a little tough to do. And so, people are going to be like, “Wind? There’s wind all the time.” And so, it has to be something else besides something like that.” —Fresno County

### 3.3. Research Question 3: What Limitations/Challenges do Public Health Agencies See with Communicating Climate Risk to their Local Communities? 

Participants pointed towards two themes: (a) the uncertainty inherent in accurately diagnosing Valley Fever—making it a challenge to disseminate clear, simple Valley Fever risk messages—and (b) the politically conservative target audience that is unreceptive to Valley Fever climate messages.

#### 3.3.1. Uncertainty Inherent to Valley Fever Diagnosis Presents Prevention Challenge

There is still so much that is unknown about Valley Fever, how it is exposed, the connection between climate, and how to prevent it. All the counties discussed how there was not a real action piece involved for preventing Valley Fever. For Kern County, this is where they saw flu and Valley Fever messaging diverge. For flu, they advertise to come get your flu shot. Tulare County echoed the same sentiments. 

“But now, we’re kind of in that last piece where there’s not a lot of action we can have people take and behavior change they can do because if your job is an outside job and you have to work, then there’s going to be exposure that happens.” —Kern County

“A lot of people don’t want to wear a mask all day long if they’re working outside. It’s very hot here during the valley fever risk period. You can’t really tell people, ‘Just sit inside all day.’ So, it’s very hard to— I mean, some of the effective strategies are, if you’re disturbing dirt, to maybe wet it down ahead of time. But we were in a really long-term drought and water usage was restricted during that time. So, it depends. There’s limited really good preventative measures for valley fever at this point.” —Tulare County

#### 3.3.2. Political Considerations in Valley Fever Messaging to Farmers

The relationship of Valley Fever fungi to climate also has a political connotation. With Fresno County, the representative spoke about how the topic of climate change with farmers is too abstract with them. The message hits a dead wall. Kings County also saw this as a limitation. 

“And then they’re not buying in, politics, right? Because they’re not buying into climate—a lot of places aren’t buying into climate. The coastal counties, coastal areas, they’re buying in. Your value areas that tend to be more conservative are not buying into climate change. So, it’s going to be—I would say we have the difficult spot of education. And a lot of people need to, hopefully—recent events, with the freezes and things, maybe that’s, right there, a pretty good little indicator of climate change, right? Man, they never had these freezes like they’re having there now. So, we’ll see what happens. A lot of it— it’s a challenge, and I’m sure you’re running into that. With climate now, it’s a big challenge. Bay area, places that are a little more open to understand it more. Or you get into areas that are more conservative that they don’t.“—Kings County

Fresno County believes a better approach to relaying climate communication would be by indicating how it impacts the Farmer’s livelihood. For example, Fresno discussed how climate affects the water allocation and how the water allocation may influence Valley Fever. “I think if we had a more stable water allocation in the Central Valley that could potentially reduce the amount of Valley Fever cases” and Fresno found that this could be used to help farmers see the benefit of purchasing more expensive, imported water. 

However, on the topic of climate change, San Luis Obispo County saw it as an unnecessary addition.

“It’s not so much that there are topics we stay away from but there are really a few specific messages that we do focus in on. So, we really try to exclude a lot of information in order to have those few messages come through clearly. We really want people to understand their risk and possible diagnostic things. And I think they’ve done some study that says that you remember maybe 15% of what a doctor or a professional tells you in any given educational session. So, we really try to get into them a few key messages. If you’re coughing for more than two weeks, you should ask your health care provider about getting tested for valley fever. If you’re out and it’s windy and gusty, you should either make sure that the dust and the dirt gets watered down or you should get out of the dust and dirt. So, we don’t find that there are taboo topics in valley fever. We just find that we want to focus in on the ones that will help our population the most.” —San Luis Obispo County

## 4. Discussion

The public health agencies discussed individuals avoiding going outside when it is dusty or windy to avoid getting Valley Fever. These agencies understood that this type of health campaign is unrealistic because the risk in the environment cannot be avoided as it is often tied to occupation. To the extent possible, public health agencies craft messages that communicate behavior adaptation over behavior avoidance to encourage the adoption of any practices that may minimize acquiring Valley Fever [9]. Currently, prevention strategies by California public health agencies include encouraging any means of dust containment; strategies may include wetting soil, tilling soil more regularly, integrating policies for construction project approval that require dust containment protocols, and discussing of wearing N95 respirators during the Valley Fever season. This last strategy poses its greatest challenge under severely hot weather conditions. Despite prevention efforts, these agencies still see construction workers and farmers getting diagnosed with Valley Fever annually. 

There are four best practices for communication: (1) identify the targeted behavior; (2) develop an effective strategy for exposing people to the message; (3) take a comprehensive approach; and (4) seek routine media exposure [4,14]. For Valley Fever, public health agencies focus on encouraging community members to go to the doctor if they have symptoms. However, in sensitive populations, behavior is driven by factors other than communication, priorities are different, and what matters to the local community varies [4]. For Valley Fever, researchers need to conduct surveys and interviews to learn what matters most to the community and design communication around the results. Although the agencies expressed that the public was at an information overload, only a small portion of the information is related to Valley Fever. According to the best practices, communication efforts need to be large scale and should come from a variety of sources— “all but the kitchen sink” [4]. The longevity of communication campaigns are also fundamental. It is important for the message to be phrased in different ways and to use different outlets to get the message disseminated to the target audience [4]. Public health agencies need to simplify the language but not the content [5]. Evaluation of media messages is a best practice and it is important for health agencies to have an open dialogue with their community in order to gauge the effectiveness of their campaign [5]. For Valley Fever, seasonal messages about getting tested is not enough. It is also important to know your audience. Media campaigns designed in only English and Spanish are not enough for Central California, where there are large tribal communities located in the area. 

However, with Valley Fever, media messages are limited due to funds. The state and local governments have expressed that they struggle with support and lack of dedicated dollars for Valley Fever [50]. Inadequate resources and organizational constraints have been recognized as key constraints to effective risk communication [5]. The importance of persistence in communication efforts has also been recognized as key to overcoming barriers [14]. Effective risk communication requires sustained effort. Fischhoff mentions the scope of communication must be comprehensive enough to include policy efforts that build the resources for prevention and effective and sustained risk communication efforts [51]. Public health communication typically accounts for 1% of the health information in the media, so public health agencies need to develop media partnerships and increase the variety and source of messages [4]. A concentrated media effort is required if agencies wish to see behavior changes in communities. 

One of the at-risk communities identified by the public health agencies for Valley Fever is farmers. Similar to the information posed by Fresno County, several studies have found that communicating climate change to farmers falls flat [9,52,53,54,55,56,57,58]. Farmers have a different relationship with the environment—it is used for their livelihood. In the past, when researchers would communicate about climate change and translate that topic for farmers, the farmers were being unintentionally marginalized by that communication, which usually required farmers to make drastic changes in their traditional farming ways [53]. Farmers base their understanding on situational experiences. Their memory of past climate and bad crop seasons need to be included into the conversation [53,55]. Previous studies found that farmers were more likely to adapt to climate change when they identified risks to their own farm and bottom line, their efficacy beliefs aligned with the adaptation measures, and their remembrance of dry years [52,57,59]. Valley Fever communication to farmers could use this similar approach when communicating climate risks to Valley Fever, such as tying outbreaks to historic water allocations and seeing if uptick in Valley Fever prevention strategies correlates to the number of farming staff that have been diagnosed with Valley Fever. Economics, specifically the cost, has also been a primary driver for farmers’ choosing adaptation efforts towards Valley Fever [54,56]. For Valley Fever, Fresno County staff believed that water allocation due to drought restrictions and letting the land fallow might be correlated to uptick in Valley Fever cases. Under this untested hypothesis, spikes in Valley Fever cases could be an unintended consequence of the drought restrictions and the need for water conservation in the area. Communicating the need for farmers to intervene in this relationship, could require farmers to purchase more expensive water. When communicating Valley Fever prevention strategies to subpopulations, like farmers, the local health agency should be aware of these potential barriers to implementation and focus on a joint adaptation strategy, possibly including government financial assistance [60]. 

Air quality was discussed by public health agencies as a good example of how an agency could communicate Valley Fever risk through its relationship with climate. Previous studies have found that people, who were more inclined to change behaviors and activities that were affected by air quality, already believed they have a perceived risk to air quality, had self-protective and information seeking behavior, and had trust in the government giving the message [61,62]. Some studies found that the air quality campaigns would be more effective through local government initiatives [63,64]. If Valley Fever messaging is designed similar to air quality messages, then a potential limitation in implementation could be that those that care about Valley Fever are already practicing the preventative behavior, so a warning system may not be effective towards increasing protective behavior. A similar system for Valley Fever would help bring more advanced warning to those individuals who have a similar self-protective behavior. To make an effective Valley Fever “bad day” system, agencies need to determine the climate trigger threshold and develop new ways to get people to care first about Valley Fever and frame it around their associated risk, and then take action when the warning system is alerted. Unintended consequences of this type of alert system should also be fully explored. One air quality program in Mexico City banned driving for its community one weekday per week, based on the last digit of the license plate [65]. The goal was to decrease the number of cars driving per day, but the program found that air quality was not improved, people got around the rule by purchasing more cars [65]. An unintended consequence of a Valley Fever “bad day” could involve individuals conducting risky behaviors on a “good day” that still falls within the Valley Fever exposure season. More research should be conducted to see how risk of Valley Fever changes with the concept of a monitoring alert program. Similarly, one study looked at behavior adaptation towards heat-related health risks that focused on sleep quality and improving sleep quality, so the body could recover from intense daytime heat waves [66]. Like Valley Fever and air quality, some people are not able to stop a risky behavior, such as working on construction activities. Research should be conducted to see if there is an action or activity that a person could do to boost their immunity to Valley Fever. 

Currently adaptation to Valley Fever is minimal. Large scale transformational adaptation is difficult to implement for Valley Fever due to the uncertainties around risks, especially related to climate change, the high costs to make any large-scale changes, and a lack of acceptable behavior modification options [67]. Current research does not identify climate thresholds or climate-related risks. For public health agencies, stating that wind has a relationship with disease outbreak is not helpful. Instead, as an example, stating that wind under 5 mph is linked to increased exposure is more helpful. It provides more content for a media strategy or an alert system. Understanding climate thresholds could also be used to guide the implementation of current strategies, such as wetting down the soil during construction activities. In addition, understanding seasonal risks associated with climate could also be used in discussing various tilling adaptation practices. However, the audiences for which these adaptation strategies are developed should be included in the method development [51]. 

For Valley Fever, research needs to focus on community understanding. Research on Valley Fever is underfunded [50]. The California Department of Public Health (CDPH) is aware of the lack of funding [50]. In California, there is no broad awareness campaign or policies geared around ensuring funding for the disease. Most local agencies get predeveloped information from the CDPH or the Centers for Disease Control (CDC) [50]. These local agencies link the information to their webpages, but the visibility, longevity, and diversity of the campaign is not there. With other states in the United States also experiencing Valley Fever budget and visibility struggles, the burden of the funding should not be strictly carried by the local agencies. A statewide and federal funding program, such as from the National Institute of Health (NIH) and the CDPH, should be developed to adequately fund new surveillance activities, and understand behavioral risk and adaptation measures. 

### Limitations

This study was conducted for Central California and extrapolation to other counties or states may not be appropriate. For the study area, the findings are consistent among the counties’ health agencies. This increases the validity of the findings towards the idea of incorporating climate communication to the disease known as Valley Fever. 

## 5. Conclusions

Currently, the relationship between climate and Valley Fever is not included in risk communication. This study suggests that future researchers partner their findings with their local public health agencies to provide the climate–disease relationship in a way that can be utilized beneficially. The target audiences should also be brought into the development of the risk communication and help expand the current behavior adaptation catalogue of strategies. By connecting the research with the behavior adaptation model, this study directs communication efforts to emphasize adaptation behaviors the target audience can reasonably adopt to lower their risk for acquiring Valley Fever. 

Future research should look at evaluating the current Valley Fever messaging in California. A future study could evaluate and compare communication strategies in Arizona and California. This type of study could shed light on the struggles and success of other agencies dealing with Valley Fever and how to build capacity for sustained prevention efforts. Researchers also need to focus their efforts on expressing uncertainty between the relationship between climate and Valley Fever, without losing the trust of the local community [7].

Given their limited resources, public health agencies are in fact disseminating tailored prevention messages to at-risk groups. First, general “get tested” messages are disseminated during peak seasons of Valley Fever to a broad audience, also disseminating such campaigns in Spanish to reach a broader at-risk group. Furthermore, prevention messages are adapted for at-risk groups including construction workers, farmers, and prison populations to emphasize mitigation strategies of dust containment, wetting and tilling soils, and enforcing these behaviors with protocols integrated into policy approval processes of construction jobs. Ideal Valley Fever risk messages capitalize on heightened recognition of familiar poor air quality or red flag days as a strategy for increased attention to these prevention messages. While generic wind messages seem to be ineffective, specific wind messages indicating threshold effects stand a higher likelihood of message acceptance and compliance. Lastly, climate change is a hot button issue and the public health agencies in Central California described their communities, especially those at higher risk, as conservative. Messages and communication efforts geared around climate change would seem to be ineffective, thus requiring a different approach towards communicating a climate sensitive disease, such as Valley Fever.
